# A demethylation-driven gene signature predicts prognosis and therapeutic vulnerability in hepatocellular carcinoma

**DOI:** 10.1038/s41598-026-41443-0

**Published:** 2026-02-26

**Authors:** Zhengwei Wang, Lei Shi, Yongjian Li, Sikai Liu, Wanyin Shi

**Affiliations:** Department of Radiology, The First Affiliated Hospital of Anhui Medical University, No. 218 Jixi Road, Shushan District, Hefei, 230022 Anhui China

**Keywords:** Hepatocellular carcinoma, DNA demethylation, Prognostic signature, Immune microenvironment, Tumor mutation burden, Biomarkers, Cancer, Computational biology and bioinformatics, Oncology

## Abstract

To develop and validate a demethylation-driven gene signature for predicting HCC prognosis, immune microenvironment features, and therapeutic vulnerabilities. We integrated transcriptomic data from TCGA-LIHC (*n* = 346 tumors) and GEO-GSE112790 (*n* = 183 tumors) with a curated list of 3,743 demethylation-related genes (DRGs). Tumor-associated DRGs were identified via differential expression analysis and WGCNA. A prognostic six-gene signature was derived using univariate Cox and LASSO regression, and a risk score model was constructed by multivariate Cox analysis. Patients were stratified into high- and low-risk groups. Model performance was evaluated using Kaplan–Meier and time-dependent ROC analyses. Immune infiltration was assessed by ssGSEA, somatic mutations were profiled, and drug sensitivity was predicted via the GDSC database. G6PD expression was validated using immunohistochemistry. A six-gene prognostic signature (CEP41, SUB1, CDC20, G6PD, VPS72, SPINDOC) was established. The risk score significantly stratified patients into high- and low-risk groups with distinct overall survival (*p* < 0.001). The model showed strong predictive accuracy with AUCs ≥ 0.70 at 1, 3, and 5 years. High-risk patients exhibited enrichment in cell cycle, DNA repair, and metabolic pathways, along with an immunosuppressive microenvironment marked by regulatory T cells and myeloid-derived suppressor cells. Somatic mutation analysis revealed differential TP53 mutation frequencies between risk groups. Drug sensitivity prediction indicated that high-risk patients may respond better to agents such as Tozasertib and Navitoclax. IHC confirmed significant upregulation of G6PD in HCC tissues, supporting its role in metabolic reprogramming. This study establishes a robust, demethylation-driven six-gene signature that effectively stratifies HCC patients into distinct prognostic groups. The model integrates multi-omic insights into tumor biology and therapeutic vulnerability, providing a clinically actionable framework for personalized risk assessment and treatment planning in hepatocellular carcinoma.

**Supplementary Information:**

The online version contains supplementary material available at 10.1038/s41598-026-41443-0.

## Introduction

Hepatocellular carcinoma (HCC) is the most prevalent form of primary liver cancer and ranks among the leading causes of cancer-related death globally^1^. According to recent estimates, more than 900,000 new cases and 830,000 deaths occur annually, with the highest burden in East Asia and sub-Saharan Africa, but with incidence steadily rising in western countries as well^1,2^. Despite advances in surgical resection, liver transplantation, and loco-regional therapies, long-term outcomes remain unsatisfactory, largely due to the high recurrence rate and the limited effectiveness of systemic treatments^3^. Immunotherapy and molecularly targeted agents have offered renewed hope, yet only a subset of patients derive durable benefit, underscoring the urgent need for reliable biomarkers that can guide risk stratification and therapeutic decision-making^4^.

One of the major obstacles in HCC management is its profound heterogeneity. Patients with similar clinical stages often exhibit markedly different survival trajectories, suggesting that current staging systems fail to capture the biological complexity of the disease^5,6^. Molecular profiling efforts have revealed that hepatocarcinogenesis is not only driven by genetic alterations but also by widespread epigenetic reprogramming^7^. Aberrant DNA methylation and demethylation have emerged as key regulators of oncogene activation, tumor suppressor silencing, and chromatin remodeling^8^. Importantly, dysregulation of demethylation-associated genes is increasingly recognized as a hallmark of tumor progression, metabolic adaptation, and immune evasion^9–11^. Recent studies have further emphasized the prognostic relevance of epigenetic mechanisms and molecular signatures in hepatocellular carcinoma (HCC), including dysregulated DNA methylation, histone modifications, chromatin regulatory enzymes, and epigenetic regulators that are associated with tumor progression and poor clinical outcomes. These alterations have been shown to contribute to HCC carcinogenesis and survival prediction across multiple cohorts^12–15^. Yet, the systematic characterization of demethylation genes in HCC and their prognostic value remains incomplete, leaving a critical gap in our understanding of epigenetic contributions to liver cancer biology^7,16^.

Previous attempts to develop prognostic gene signatures in HCC have demonstrated proof-of-principle for transcriptomic-based stratification, but these models often lack mechanistic interpretability^17^, fail to integrate epigenetic and immune features^18,19^, and show limited reproducibility across independent cohorts^20^. Moreover, most signatures are not directly linked to therapeutic vulnerabilities, which diminishes their translational relevance^21^. Thus, there is a pressing need for integrative prognostic models that are not only statistically robust but also biologically informative and clinically actionable.

In this study, we performed a systematic investigation of tumor-associated demethylation genes in HCC, leveraging large-scale transcriptomic and genomic datasets. Through rigorous feature selection and multivariate modeling, we established a robust prognostic signature and validated its performance across independent cohorts. Gene Ontology (GO) and Kyoto Encyclopedia of Genes and Genomes (KEGG) pathway analysis highlighted the interplay between central carbon metabolism and DNA repair, implicating epigenetic demethylation in fundamental processes of malignant transformation. Functional enrichment analyses revealed that the identified genes converge on critical biological programs, including protein translation, metabolic reprogramming, and chromatin organization. Immune infiltration profiling further uncovered distinct immune landscapes between high- and low-risk groups, pointing to an epigenetic basis for tumor-immune crosstalk. In addition, mutational profiling and drug sensitivity prediction revealed therapeutic vulnerabilities that may inform personalized treatment strategies.

Together, our findings bridge the gap between epigenetic dysregulation and clinical heterogeneity in HCC. By linking demethylation-associated transcriptional programs to prognosis, immunity, and therapeutic sensitivity, this work provides not only a clinically relevant prognostic model but also novel biological insights into liver cancer pathogenesis. Such integrative frameworks have the potential to refine patient stratification and support the rational development of targeted and immunotherapeutic approaches, ultimately improving outcomes for patients with HCC.

## Materials and methods

### Data resources

We integrated three complementary datasets to enable both discovery and validation of demethylation-related prognostic markers in HCC. First, transcriptomic profiles and matched clinical annotations for liver hepatocellular carcinoma (TCGA-LIHC) were retrieved from the TCGA/GDC repository. The initial dataset contained 374 tumor and 50 adjacent normal tissues. To ensure robust survival modeling, we excluded cases lacking complete follow-up information and those with overall survival (OS) < 30 days, thereby minimizing perioperative or non-disease-related deaths. This yielded 346 tumor samples for prognostic analysis. Second, to provide independent external validation of expression patterns, we obtained the GEO series GSE112790, which contains 183 tumor and 15 adjacent normal liver samples. This dataset was used to validate the differential expression of candidate prognostic genes identified in the TCGA training cohort. Third, a curated list of demethylation-related genes (DRGs) was obtained from the GeneCards database by querying the keyword “demethylation”. At the time of retrieval, a total of 3,743 genes annotated as being associated with demethylation were collected and used for downstream analyses. The complete list of these genes is provided in Supplementary Table S1. This DRG set was intersected with the processed expression matrices from TCGA and GEO to define the candidate space for downstream feature selection and survival modeling. As all datasets were obtained from public repositories, no additional institutional ethics review was required for this secondary analysis. This retrospective study approved by the ethics committees of The First Affiliated Hospital of Anhui Medical University (Research Ethics No: PJ2025-08–61).

### RNA-seq data quality control, normalization and differential expression analysis

We developed a reproducible analysis pipeline to generate standardized expression matrices for discovery and validation. For TCGA data, transcriptome, clinical, and survival tables were merged by sample identifier, with tumor and normal status annotated accordingly. Ensemble gene IDs were mapped to gene symbols using GENCODE v36; when multiple probes mapped to the same gene, the probe with the highest mean expression was retained. Genes lacking annotation and low-abundance genes (counts per million < 1 in > 50% of samples) were excluded. To account for library size and compositional biases, TMM normalization was applied (edgeR), followed by voom transformation to obtain log2-CPM values with precision weights. Between-array normalization was applied where necessary. The GEO validation cohort was processed analogously after probe re-annotation and collapse. Differential expression analysis was performed with limma, using a design matrix encoding tissue type and applying empirical Bayes moderation. Differentially expressed genes (DEGs) were defined as those with FDR-adjusted *P* < 0.05 and |log2 fold change| > 1, corresponding to an approximate twofold change in expression. Four standardized outputs were generated for downstream analyses: normalized log2-CPM expression matrix, clinical metadata, tumor/normal labels, and curated survival data. These served as the foundation for feature selection and prognostic modeling.

### Functional enrichment analysis of demethylation-related DEGs

To elucidate the biological roles and pathway contexts of demethylation-related genes differentially expressed in HCC, we performed functional enrichment analyses using the ClusterProfiler package in R. GO analysis was conducted across the three major domains, namely biological process (BP), cellular component (CC), and molecular function (MF), with particular emphasis on BP terms to capture the biological mechanisms underlying tumor progression. KEGG pathway analysis was carried out in parallel to identify metabolic, signaling, and disease-associated pathways enriched among these genes. Enrichment significance was assessed using Benjamini-Hochberg adjusted P values, and terms with adj.P.value < 0.05 were considered statistically significant. This integrative approach provided a systematic interpretation of the functional landscape and signaling networks associated with the identified demethylation-related DEGs.

### Weighted gene co-expression network analysis

Weighted gene co-expression network analysis (WGCNA) was applied to identify gene modules with coordinated expression patterns and to investigate their associations with clinical traits in HCC. Genes with low abundance (mean FPKM ≤ 0.5) were excluded, and sample quality was evaluated using the goodSamplesGenes function. Hierarchical clustering (hclust) was performed to detect and remove outlier samples. To establish a scale-free network, we calculated the scale-free topology fit index and mean connectivity across a range of soft-threshold powers, and a power of 15 was selected since it achieved a fit index R² greater than 0.8. The adjacency matrix was subsequently transformed into a topological overlap matrix to quantify network interconnectedness. Gene modules were identified using dynamic tree cutting with a minimum module size of 100 genes, and modules with eigengene correlations greater than 0.9 were merged. Pearson correlation analysis between module eigengenes (MEs) and clinical traits was then performed to determine trait-associated modules. Among them, the blue module demonstrated the strongest and most significant correlation with tumor status. For the identification of key genes, we calculated module membership (MM) and gene significance (GS) values, visualized MM-GS correlations for each module, and integrated module assignments together with MM and GS values into an annotated gene list for downstream analyses.

### Identification of prognostic demethylation-related genes and model interpretability

To identify HCC-associated demethylation-related genes with prognostic relevance, we integrated multiple analytical strategies. First, we intersected three gene sets: DEGs, WGCNA key module genes, and the curated demethylation-related gene list. Overlaps were visualized and quantified using the ggvenn package. To derive core prognostic markers, the TCGA-LIHC cohort was randomly divided into a training set (70%) and an internal testing set (30%) using stratified randomization based on overall survival status to ensure baseline consistency between the two groups. For external validation, we downloaded the HCC transcriptome dataset GSE76427 from the GEO and applied the established prognostic model to the 95 tumor samples with complete survival information, computing risk scores, Kaplan–Meier survival curves, and time‑dependent ROC curves to assess predictive performance. Univariate Cox proportional hazards regression was applied in the training set to screen genes significantly associated with OS. Candidate genes were further refined using LASSO regression with L1 regularization to reduce redundancy and enhance model sparsity. The selected genes were then incorporated into a multivariate Cox regression model to construct a prognostic signature. A patient-specific risk score was calculated as a weighted sum of gene expression values, and patients were stratified into high- and low-risk groups based on the median risk score in the training cohort. To enhance model interpretability, SHapley Additive exPlanations (SHAP) analysis was applied to the multivariate Cox model, quantifying the contribution of each gene to predicted risk in the test set.

### Survival analysis and prognostic nomogram construction

The prognostic value of the Cox-derived risk score was assessed by comparing OS between high- and low-risk groups in both training and test cohorts using Kaplan-Meier estimation with two-sided log-rank tests. Survival curves were generated with the ggsurvplot function, with extreme significance indicated for *P* < 0.001. Separation of survival curves and median survival times was used to evaluate the discriminatory power of risk stratification. To facilitate clinical application, a prognostic nomogram was constructed by integrating the risk score with independent clinical covariates identified via multivariate cox regression. The nomogram was generated using the regplot function, with each predictor assigned a weighted score proportional to its hazard ratio, enabling individualized prediction of 1-, 3-, and 5-year OS probabilities. Calibration was assessed using bootstrap resampling to generate calibration plots comparing predicted versus observed survival. Discriminatory performance was further evaluated using time-dependent receiver operating characteristic (ROC) curves computed with the timeROC package, with areas under the curve (AUC) for 1-, 3-, and 5-year OS calculated. AUC values greater than 0.7 were considered indicative of good predictive performance. These analyses collectively provide a robust and interpretable framework for patient-specific survival estimation in HCC.

### Validation, functional annotation, and immune infiltration analysis

To confirm the differential expression and prognostic relevance of candidate genes, expression levels were compared between tumor and adjacent normal tissues in both the internal testing cohort and the independent external validation set using the Wilcoxon rank-sum test. This approach ensured the consistency and robustness of candidate biomarkers across datasets. To explore underlying biological mechanisms, Gene Set Enrichment Analysis (GSEA) was performed based on Spearman correlations between each biomarker and genome-wide expression profiles. KEGG pathway enrichment was conducted using the clusterProfiler R package with an adjusted P value cutoff of 0.05. The top five enriched pathways, ranked by absolute normalized enrichment score (NES), were visualized to highlight key processes potentially driving tumor progression or treatment response. Given the critical role of the tumor immune microenvironment, single-sample GSEA (ssGSEA) was applied to estimate immune cell infiltration and immune-related functional signatures across risk groups. Comparisons of immune scores between high- and low-risk patients revealed distinct immune landscape patterns associated with prognosis. Additionally, pan-cancer expression profiles of prognostic genes were examined using the GSCA platform to evaluate their relevance and specificity across diverse malignancies.

### Somatic mutation profiling and drug sensitivity prediction

To characterize the genomic landscape underlying differential prognosis in HCC, somatic mutation data were obtained from TCGA. Tumor mutation burden (TMB) was calculated for each patient as the total number of nonsynonymous mutations per megabase, serving as a proxy for neoantigen load and potential immunotherapy response. Mutation profiles were visualized using the maftools package, generating oncoplots summarizing mutation frequencies, variant types, and gene-level alterations stratified by high- and low-risk groups. Comparative analysis of mutation spectra enabled identification of significantly mutated genes and distinct mutational signatures associated with risk stratification, providing insights into tumor heterogeneity and evolution. In parallel, chemotherapeutic sensitivity was predicted by integrating gene expression data with pharmacogenomic profiles from the Genomics of Drug Sensitivity in Cancer (GDSC) database. The pRRophetic algorithm was used to estimate half-maximal inhibitory concentration (IC50) values for a panel of anticancer drugs in each HCC sample. Differences in predicted IC50 values between high- and low-risk groups were assessed using the Wilcoxon rank-sum test, identifying candidate drugs with differential efficacy to guide precision therapy. Drug sensitivity prediction was performed using the pRRophetic R package (PMID: 25229481), which applies pre-trained ridge regression models built from baseline gene expression and drug response data in large cancer cell line panels. These models have been previously validated to meaningfully estimate drug response when applied to external datasets, and in this study were used to generate predicted IC50 values that were compared between high- and low-risk groups. This integrated mutational and pharmacogenomic analysis complements prognostic modeling and provides a translational framework for clinical decision-making in HCC.

### Immunohistochemistry

Immunohistochemistry (IHC) was performed on formalin-fixed, paraffin-embedded (FFPE) Sect. (4 μm). After deparaffinization, antigen retrieval was achieved in citrate buffer (pH 6.0). Endogenous peroxidase activity was blocked with 3% hydrogen peroxide, followed by incubation with 5% BSA. Sections were incubated overnight at 4 °C with primary antibodies against [G6PD,1:100, proteintech]. Detection was performed using HRP-conjugated secondary antibodies and DAB substrate, with hematoxylin counterstaining. Staining intensity and percentage of positive cells were independently evaluated by two blinded pathologists, and semi-quantitative scores were obtained.

### Statistical analysis

All statistical analyses were conducted in R (version 4.2.2) unless otherwise specified. Continuous variables were compared using the Wilcoxon rank-sum test for nonparametric distributions. Survival differences were evaluated using Kaplan-Meier estimation with two-sided log-rank tests. Cox proportional hazards regression models were employed for univariate and multivariate survival analyses, with proportionality assumptions verified using Schoenfeld residuals. Multiple testing corrections were applied using the Benjamini-Hochberg method to control the false discovery rate. A two-tailed P value < 0.05 was considered statistically significant unless stated otherwise. Data visualization was implemented using the ggplot2, survminer, and pheatmap packages.

## Results

### Systems-level transcriptomic and functional analysis identifies tumor-associated demethylation genes

**Fig. 1:**
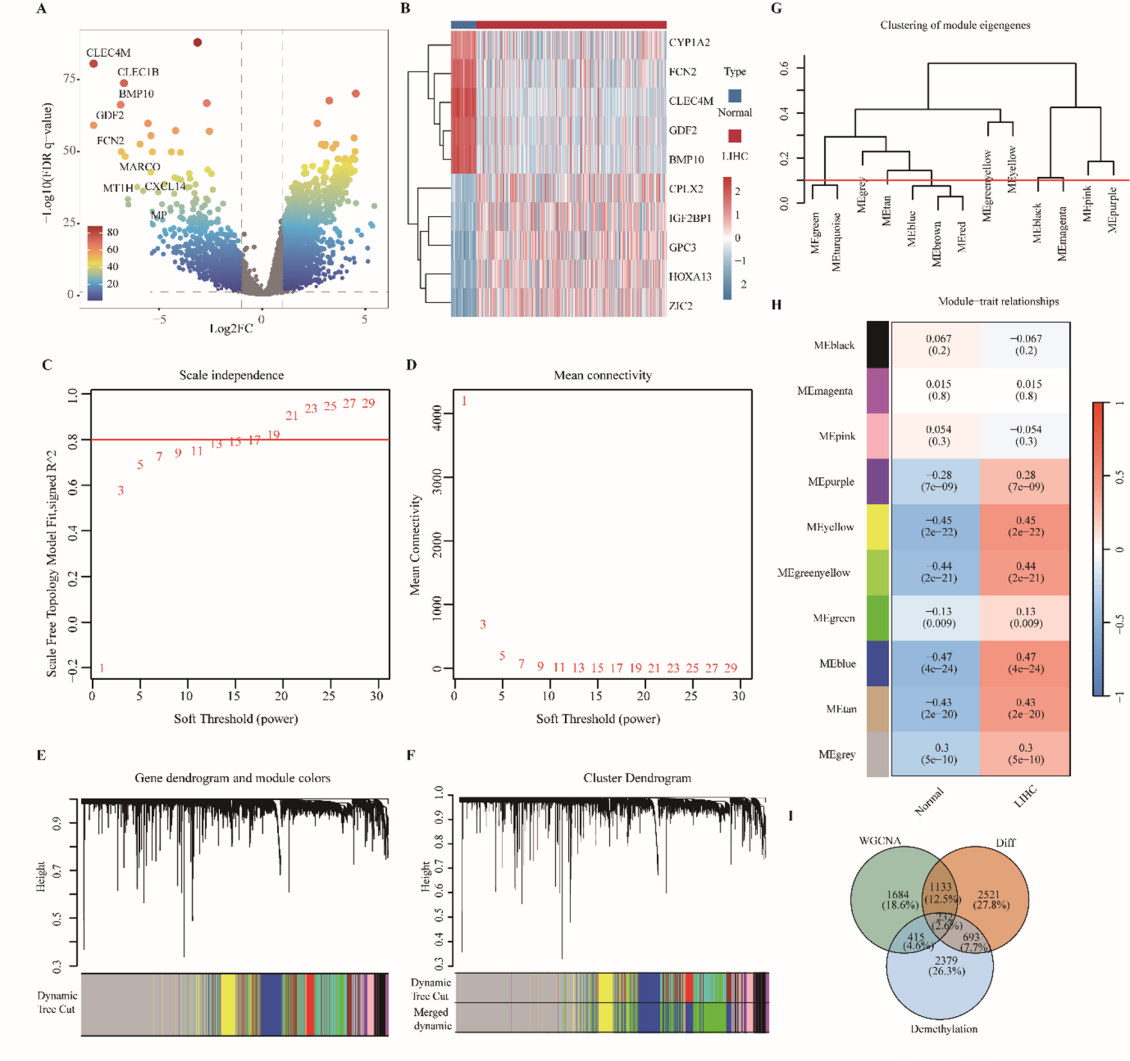
Systems-level differential expression and weighted gene co-expression network analysis identify tumour-associated demethylation genes. (**A**) Volcano plot of differential expression between tumour and adjacent normal liver; x-axis, log₂ fold change; y-axis, −log₁₀(adjusted P); color and size indicate fold change direction/magnitude and statistical significance. Dashed lines mark |log₂FC| = 1 and significance threshold. (**B**) Heat map of differentially expressed genes; columns = samples, rows = genes; red-blue scale indicates expression (red = high, blue = low). Annotation bar shows sample group (normal = blue, tumour = red). (**C-D**) Soft-threshold selection: (**C**) scale-free topology fit index; (**D**) mean connectivity versus candidate powers (β) to guide network construction. (**E**) Gene dendrogram from hierarchical clustering with dynamic tree cutting; colored strip indicates module assignment. (**F-G**) Module eigengene clustering and merging (**F**) and final dendrogram with merged module colors (**G**). (**H**) Module-trait correlation heat map; rows = modules, columns = clinical traits; color indicates Pearson correlation (red = positive, blue = negative); each cell reports correlation coefficient and P value. (**I**) Venn diagram showing intersection of blue-module hub genes, differentially expressed genes, and literature-curated demethylation gene set, yielding tumour-associated demethylation gene candidates.

**Fig. 2:**
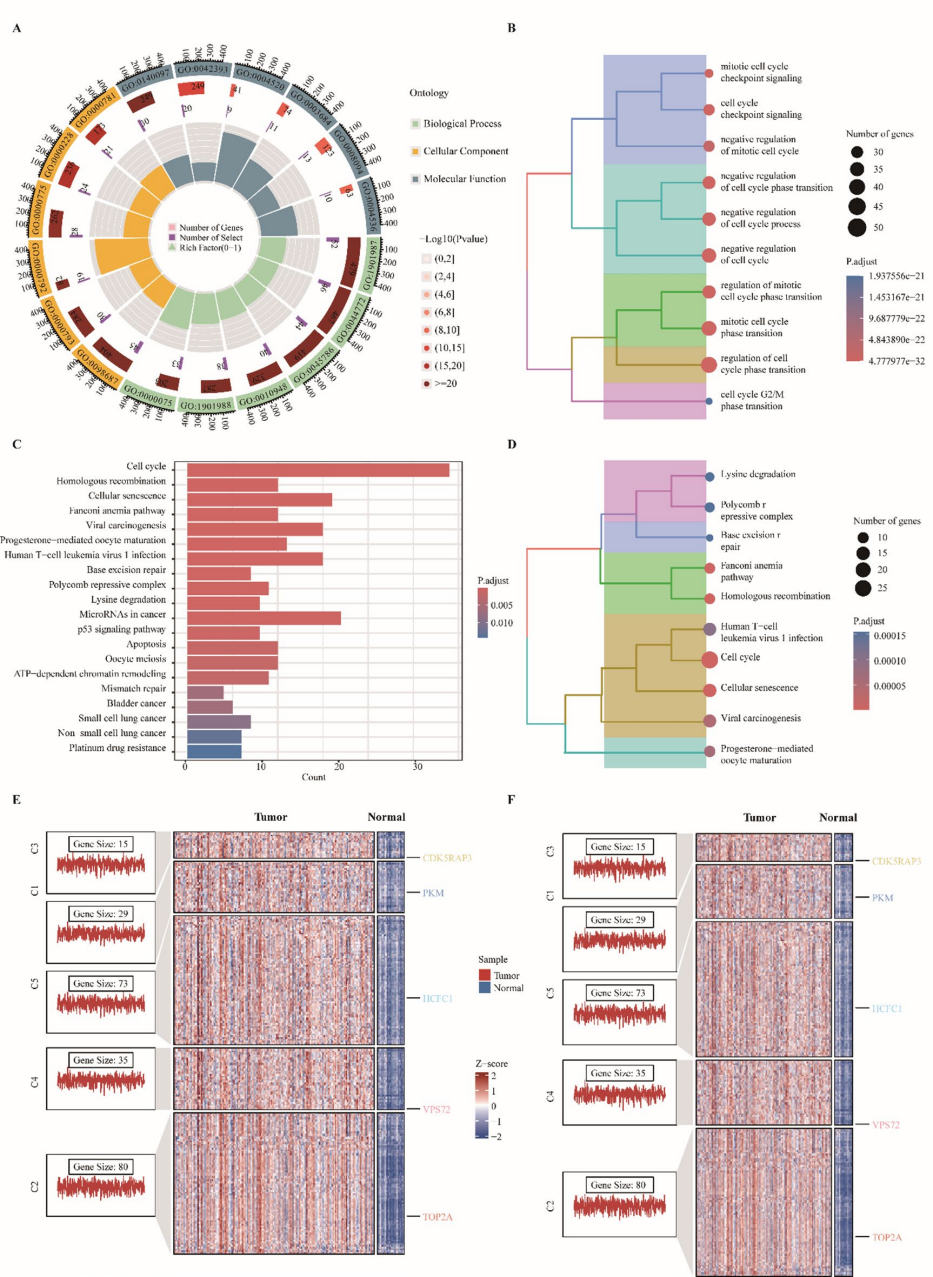
Functional enrichment and expression-module analysis of tumour-associated demethylation genes. (**A**) Circular summary of Gene Ontology (GO) enrichment. Tracks show GO categories (BP, CC, MF), matched gene counts, enriched gene counts (color intensity = enrichment magnitude), and GO term IDs. (**B**) GO enrichment clustering tree; node proximity reflects term similarity, node color indicates significance, and node size indicates annotated gene count. (**C**) KEGG enrichment bar plot; x-axis = number of enriched genes, color = adjusted P value. (**D**) KEGG enrichment clustering tree; node proximity reflects pathway similarity, node color = significance, node size = pathway gene count. (**E**) Expression-module clustering (GO-centric). Five gene modules (C1-C5) shown with sample-gene heat map (Z-score normalized, red = high, blue = low), representative module genes, and top GO Biological Process terms per module. (**F**) Expression-module clustering (KEGG-centric). Same modules shown with gene expression heat map, representative genes, and principal KEGG pathways enriched per module.

Analysis of TCGA-LIHC revealed pervasive transcriptional reprogramming: 4,579 genes were differentially expressed between tumors and matched adjacent normal tissues (adj. *P* < 0.05, |log₂FC| > 1), with 3,235 upregulated and 1,344 downregulated transcripts, indicating a tumor-biased activation program (Fig. 1A). A compact tumor-specific signature was exemplified by consistently upregulated genes (CYP1A2, FCN2, CLEC4M, GDF2, BMP10) and downregulated genes (CPLX2, IGF2BP1, GPC3, HOXA13, ZIC2) (Fig. 1B). To resolve higher-order structure, we constructed a weighted co-expression network and selected a soft-threshold power of β = 15 to achieve scale-free topology (R² of approximately 0.8), yielding ten stable modules (Fig. 1C-G). Module-trait correlation identified a single tumor-linked module (blue) with the strongest positive association to tumor status (Fig. 1H, *P* < 0.001). Intersection of blue-module hubs (*n* = 3,464), the 4,579 DEGs and a curated list of 3,743 demethylation-related genes produced a focused set of 232 tumor-associated demethylation genes (Fig. 1I). Functional enrichment of the 232 tumor-associated demethylation genes revealed a coherent signal linking epigenetic deregulation to metabolic rewiring and genome maintenance. GO analysis returned 591 significant terms (426 BP, 66 CC, 99 MF), with dominant signals in translation, protein homeostasis and DNA repair (Fig. 2A-B). Key biological programs included positive regulation of cytoplasmic translation, protein stabilization and homologous recombination-mediated double-strand break repair, implicating coordinated control of protein synthesis and DNA-damage tolerance in HCC. KEGG analysis identified 23 enriched pathways that grouped into discrete functional modules (Fig. 2C-D), among the most prominent were pyruvate metabolism, cobalamin transport and metabolism, and ATP-dependent chromatin remodeling, highlighting a mechanistic link between central carbon metabolism and chromatin dynamics. Unsupervised mfuzz clustering partitioned expression space into five reproducible clusters (C1-C5), each showing tumor-biased upregulation (Fig. 2E-F), metabolism-enriched modules were dominated by metabolic enzymes, whereas DNA-repair/chromatin modules comprised genome-stability regulators. These results indicate that demethylation-associated transcriptional changes concentrate on two principal axes, metabolic reprogramming and chromatin remodeling, warranting focused functional and prognostic follow-up.

### Core prognostic genes enable survival prediction and molecular risk stratification

**Fig. 3:**
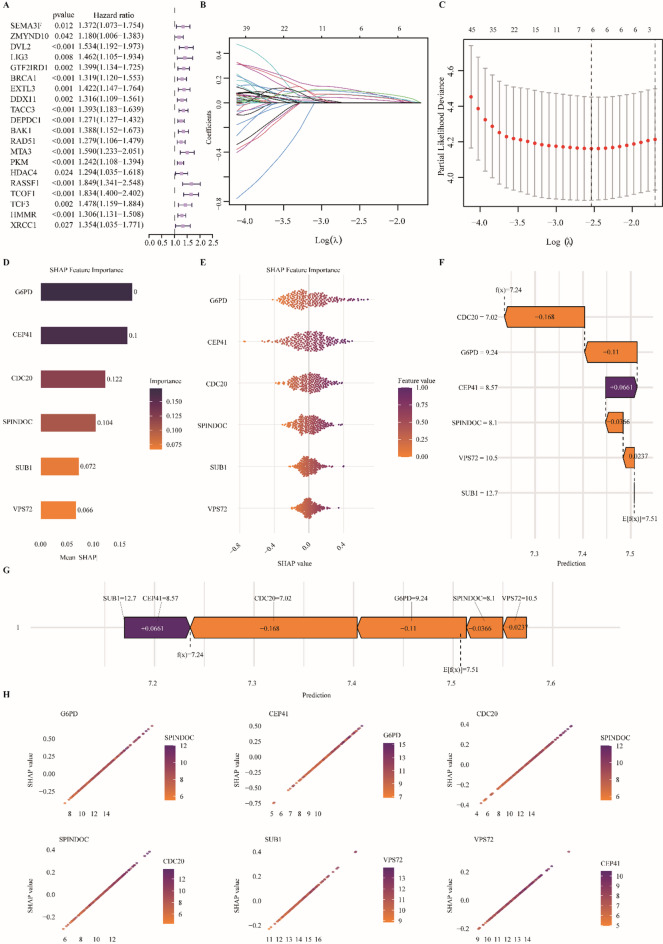
Feature selection and model interpretability for the prognostic signature. (**A**) Univariate cox forest plot showing hazard ratios (points) with 95% CIs (horizontal lines); red = HR > 1 (risk), green = HR < 1 (protective); P values listed. (**B**) LASSO coefficient path: x = L1 norm (model complexity), y = coefficient values; curves show coefficient shrinkage with increasing penalty. (**C**) LASSO cross-validation: log(λ) versus partial likelihood deviance; vertical lines mark λ_min and λ_1se. (**D**) Global feature importance: mean absolute SHAP values ranked by contribution. (**E**) SHAP beeswarm: each point = sample; x = SHAP value (effect on prediction), color = feature value. (**F**) Waterfall plot for a single sample: baseline E[f(x)] and cumulative feature contributions (yellow = increase risk; purple = decrease risk). (**G**) SHAP force plot summarizing positive and negative feature contributions to a prediction. (**H**) SHAP dependence plot: feature expression (x) versus SHAP value (y); point color denotes interacting feature; SHAP > 0 raises predicted risk.

**Fig. 4:**
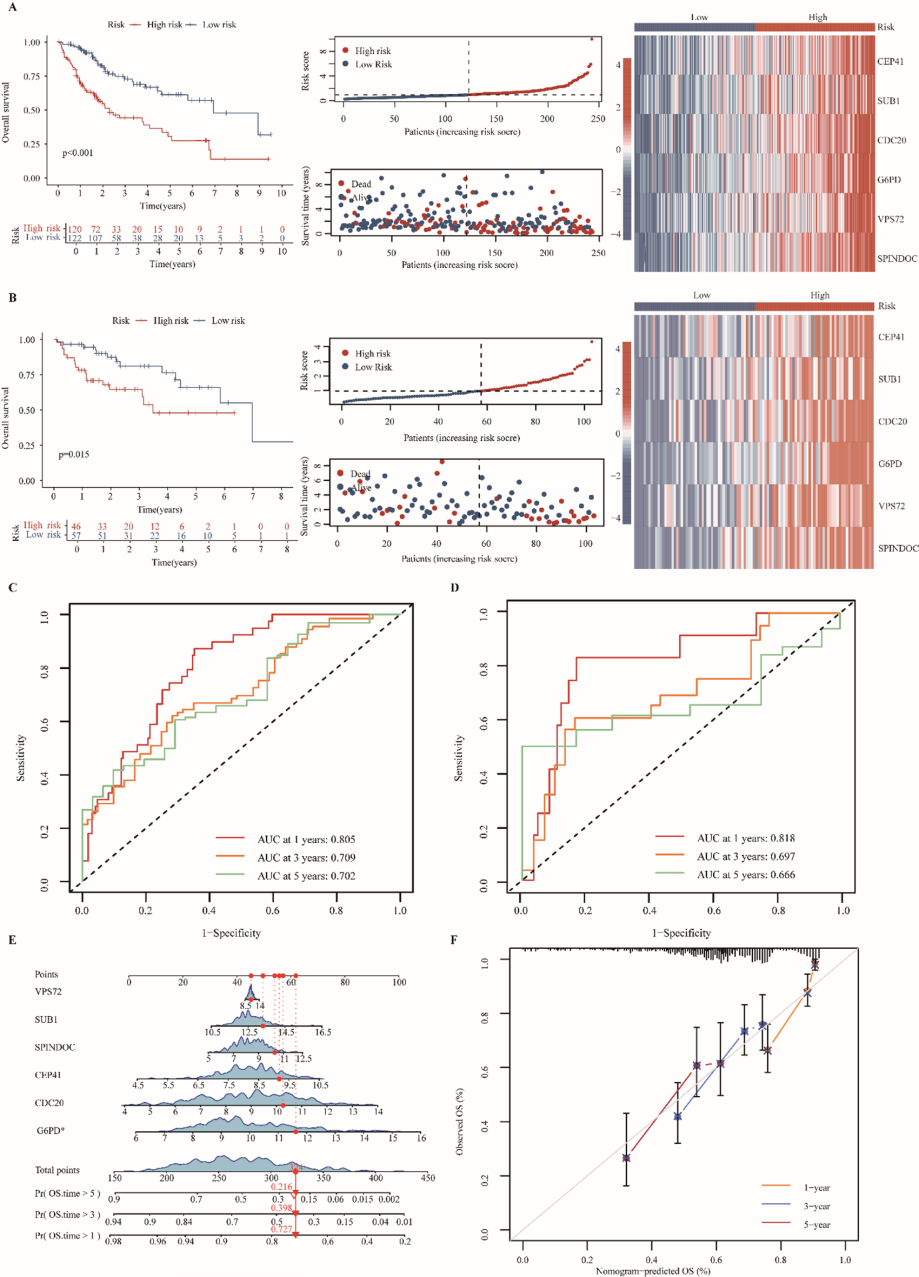
Evaluation of the prognostic gene signature. (**A**) Training cohort: Kaplan-Meier survival analysis comparing overall survival between high- and low-risk groups. Red and blue curves represent high- and low-risk patients, respectively. Patients were stratified by risk score and arranged in ascending order. The scatterplot shows survival status (red: deceased, blue: alive), with more deaths observed in the high-risk group. Heatmap illustrates the expression profiles of prognostic genes across risk groups. (**B**) Testing cohort: Kaplan-Meier curves, risk distribution, survival status, and heatmap, analyzed as in the training set. (**C**) ROC curves in the training cohort for 1-, 3-, and 5-year survival prediction. (**D**) ROC curves in the testing cohort for 1-, 3-, and 5-year survival prediction. (**E**) Nomogram predicting 1-, 3-, and 5-year survival. Each gene contributes points proportional to its prognostic weight; total points correspond to survival probability at different timepoints (red line: example calculation). (**F**) Calibration curves assessing the agreement between nomogram-predicted and observed survival probabilities at 1, 3, and 5 years. Lines closer to the diagonal indicate better consistency.

To identify a minimal gene set with robust prognostic value, we applied univariate Cox regression to the 232 tumor-associated demethylation genes in the TCGA training cohort. This analysis identified 192 genes significantly associated with overall survival in Fig. 3A (*P* < 0.05), indicating a broad transcriptional signature with prognostic relevance. Lasso penalized regression further refined this set by eliminating genes with coefficients shrunk to zero, yielding six core prognostic genes: CEP41, SUB1, CDC20, G6PD, VPS72, and SPINDOC (Fig. 3B-C). We next constructed a multivariate Cox regression model integrating these six genes into a composite risk score. SHAP analysis confirmed their collective predictive contribution, with G6PD showing the highest global importance, and elevated expression of all six genes consistently increasing predicted risk (Fig. 3D-H). When applied to the full TCGA cohort, the six-gene model stratified patients into high- and low-risk groups by median risk score. Expression analysis revealed significantly higher expression of all six genes in the high-risk group (Fig. 4A-B), and survival curves demonstrated that mortality events clustered predominantly among high-risk patients. Time-dependent ROC analysis validated the model’s predictive performance, yielding AUCs of 0.805, 0.709, and 0.702 at 1, 3, and 5 years in the training cohort, and 0.818, 0.697, and 0.666 in the test cohort, respectively (Fig. 4C-D). Nomogram construction further illustrated the additive contribution of each gene to individualized survival predictions at 1, 3, 5, and 10 years, with calibration curves demonstrating close concordance between predicted and observed outcomes (Fig. 4E-F). Kaplan-Meier survival analysis of each gene individually confirmed significant associations with overall survival in both training and test cohorts (*P* < 0.05; Supplementary Fig. S1A), underscoring their potential as independent prognostic biomarkers. Moreover, correlation analysis revealed strong associations between each gene and the composite risk score (|R| > 0.6; Supplementary Fig. S1B), indicating that these six genes constitute the principal drivers of patient stratification and underpin the predictive power of the model. For clarity, the correlation analyses between each core gene and the composite risk score were performed separately in the training and test cohorts, and the reported |R| values represent the minimum correlation observed across the two datasets. Furthermore, the prognostic performance of the six-gene risk model was externally validated in an independent HCC cohort (GSE76427, *n* = 95). Using the same risk score formula derived from the TCGA training cohort, patients in the external dataset were stratified into high- and low-risk groups. The model consistently reproduced significant survival differences, with corresponding Kaplan–Meier survival curves, risk score distributions, survival status plots, heatmaps, and time-dependent ROC analyses shown in Supplementary Fig. S2.

### Prognostic genes shape tumor progression and reprogram the immune microenvironment

**Fig. 5:**
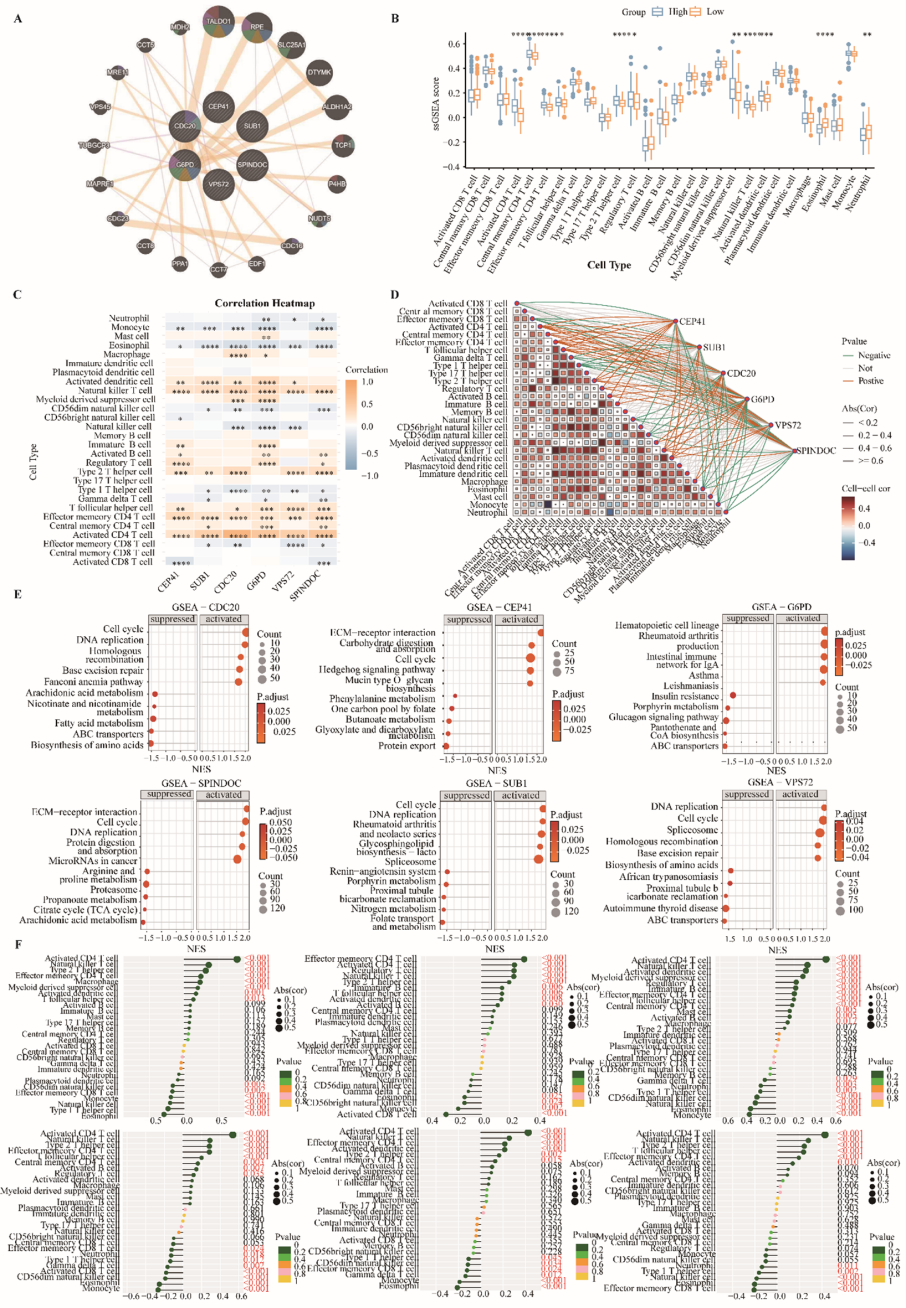
Functional and immune relevance of prognostic genes. (**A**) GeneMANIA interaction network of prognostic genes. Inner nodes represent prognostic genes, while outer nodes indicate predicted related genes. Edges denote functional interactions, and colored segments within nodes highlight enriched functions. (**B**) ssGSEA-based immune infiltration. Boxplots compare ssGSEA scores of different immune cell types between low- and high-risk groups. Statistical significance is indicated by *p < 0.05, **p < 0.01, ***p < 0.001, ****p < 0.0001. (**C**) Heatmap of correlations between immune cells and prognostic genes. Color intensity reflects correlation strength (blue: negative; orange: positive). (**D**) Correlation network between immune cells and prognostic genes. Edge thickness represents correlation strength; green and orange denote negative and positive correlations, respectively. In the immune-cell correlation subnetwork, red indicates stronger positive correlation, blue indicates stronger negative correlation. (**E**) GSEA enrichment bar plot of individual prognostic genes. X-axis shows normalized enrichment score (NES), Y-axis lists enriched pathways. Dot size represents gene count, and color reflects adjusted p value. Left bars denote suppressed pathways, right bars indicate activated pathways. (**F**) Correlation of individual prognostic genes with immune cell abundance. Larger dots indicate stronger correlation; dots on the left of the axis denote negative correlation, and those on the right indicate positive correlation.

To delineate the biological context of the six core prognostic genes (CEP41, SUB1, CDC20, G6PD, VPS72, and SPINDOC) and their associated networks, we first applied GeneMANIA, which identified 20 functionally related partners, including TALDO1, RPE, SLC25A1, DTYMK, ALDH1A2, TCP1, P4HB, NUDT5, CDC16, EDF1, CCT7, PPA1, CCT8, CDC23, MAPRE1, TUBGCP3, VPS45, MRE11, CCT5, and MDH2 (Fig. 5A). Functional annotation indicated that these genes converge on nuclear chromosome segregation, regulation of chromosome organization, pentose and NADP metabolism, monosaccharide processing, NADPH regeneration, and cellular response to interleukin-12, highlighting a coordinated role in chromosomal dynamics and metabolic adaptation in HCC. To further investigate the tumor immune landscape, we employed ssGSEA to quantify the infiltration of 28 immune cell types. Stratification by risk score revealed marked differences between high- and low-risk groups, particularly in activated CD4 T cells, central memory CD4 T cells, effector memory CD4 T cells, T follicular helper cells, type 2 T helper cells, regulatory T cells, myeloid-derived suppressor cells, and eosinophils (Fig. 5B-D). Except for neutrophils and eosinophils, which were enriched in the low-risk group, most immune cell subsets were elevated in high-risk patients, suggesting a more immunosuppressive tumor microenvironment that may underlie resistance to monotherapy. Single-gene GSEA further demonstrated that CEP41, SUB1, CDC20, G6PD, VPS72, and SPINDOC are significantly enriched in pathways related to cell cycle regulation, DNA replication, p53 signaling, and metabolic reprogramming (Fig. 5E), supporting their mechanistic involvement in tumor progression. Consistently, correlation analyses revealed robust associations between individual prognostic genes and immune cell infiltration across multiple lineages (Fig. 5F). Spearman correlation further confirmed that activated CD4 T cells and natural killer T cells positively correlated with the risk score (*R* > 0.3; Supplementary Fig. S3), suggesting that these immune populations are preferentially enriched or activated in patients with poor prognosis.

### ceRNA networks modulate core prognostic genes with conserved dysregulation across cancer types

**Fig. 6:**
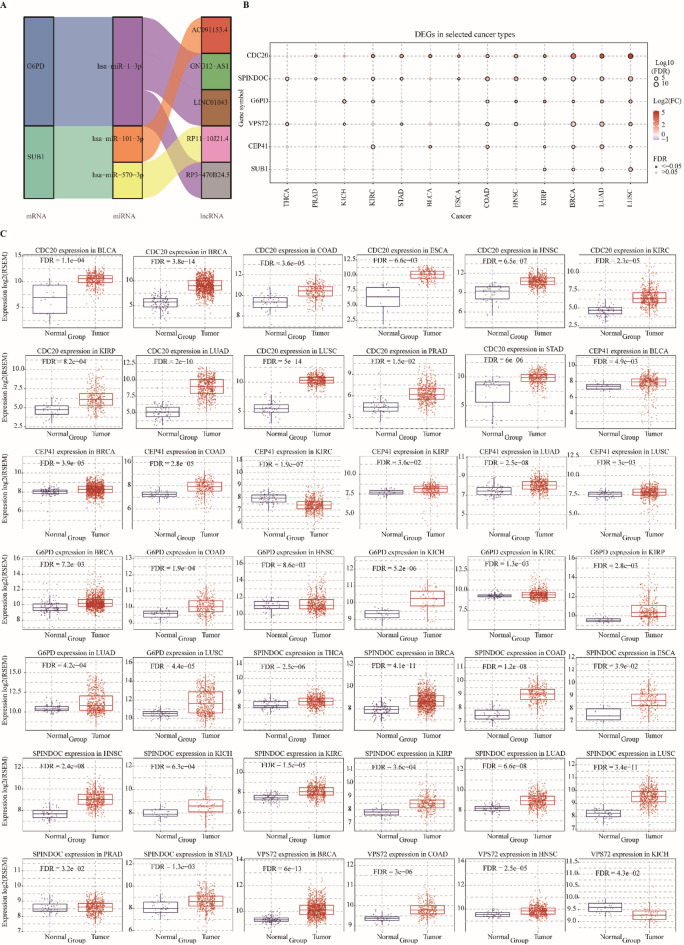
Key prognostic gene regulatory network and pancancer expression analysis. (**A**) Sankey diagram of the ceRNA regulatory network. Left nodes represent mRNAs, middle nodes represent miRNAs, and right nodes represent lncRNAs. Different colors of the links indicate different types of regulatory interactions. (**B**) Expression profiles of key prognostic genes across 13 cancer types. Each box/violin plot shows the distribution of gene expression, with significance indicated (*p* < 0.05). (**C**) Pancancer analysis of key prognostic genes in additional cancer types (*p* < 0.05). Only cancer types showing statistically significant differential expression are displayed; colors/symbols indicate up- or down-regulation. Statistical tests and multiple testing corrections are detailed in the Methods section.

To investigate potential post-transcriptional regulation of the six core prognostic genes, we constructed a ceRNA network integrating multiple databases. Database-specific default high-confidence thresholds were applied to minimize false-positive predictions. miRNA prediction using miRanda, miRDB, miRTarBase, and TargetScan identified three candidate miRNAs, while lncRNA targets predicted by spongeScan revealed five putative lncRNAs. Together, these formed regulatory lncRNA-miRNA-mRNA axes, visualized as a Sankey diagram (Fig. 6A), suggesting that ceRNA interactions may fine-tune the activity of prognostic genes and contribute to HCC pathogenesis. We next examined whether these genes are similarly dysregulated across other malignancies using the GSCA database. Thirteen tumor types with sufficient paired samples were analyzed, including BRCA, PRAD, KICH, STAD, ESCA, THCA, BLCA, KIRP, HNSC, COAD, LUAD, LUSC, and KIRC. CDC20 was consistently upregulated in BLCA, BRCA, COAD, ESCA, HNSC, KIRC, KIRP, LUAD, LUSC, PRAD, and STAD. CEP41 was elevated in BLCA, BRCA, COAD, LUAD, LUSC, and KIRP. G6PD expression was increased in BRCA, COAD, HNSC, KICH, KIRC, KIRP, LUAD, and LUSC. SPINDOC exhibited broad upregulation across all 13 cancers analyzed, including BRCA, COAD, ESCA, HNSC, KICH, KIRC, KIRP, LUAD, LUSC, PRAD, STAD, THCA, and BLCA. SUB1 was overexpressed in BRCA, KIRP, LUAD, and LUSC, while VPS72 was elevated in BRCA, COAD, HNSC, KICH, LUAD, LUSC, STAD, and THCA (Fig. 6B-D). Collectively, these results indicate that the prognostic genes identified in HCC are not only embedded within ceRNA-mediated regulatory circuits but also display conserved patterns of dysregulation across diverse malignancies, underscoring their potential as broadly relevant biomarkers in cancer.

### High-risk HCC patients exhibit distinct somatic mutation profiles and differential drug sensitivity

**Fig. 7:**
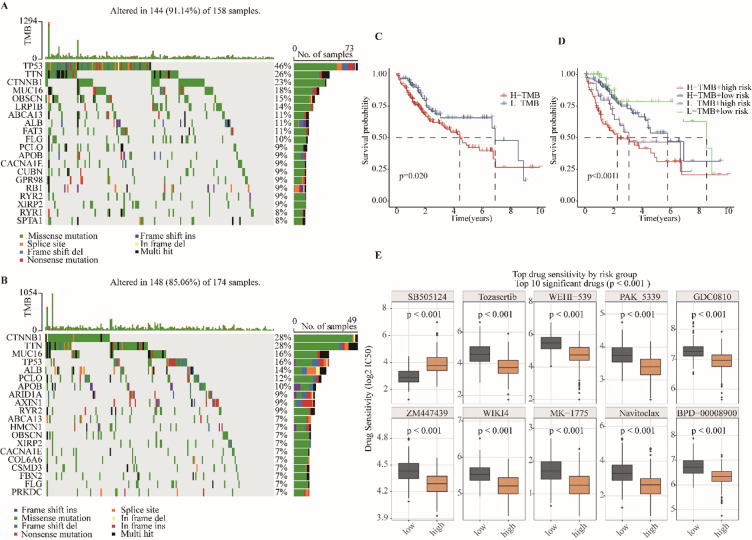
Somatic mutation landscape and survival analysis in high- and low-risk groups. (**A-B**) Somatic mutations in high-risk (**A**) and low-risk (**B**) tumors. Each row represents a gene and each column a sample. Bar colors indicate mutation types; the right-side bar plot shows mutation frequencies. Frame Shift Del/Ins: Deletion/insertion causing a frameshift, often producing truncated or nonfunctional proteins. Missense: Single nucleotide change leading to amino acid substitution. Nonsense: Point mutation generating a premature stop codon. Multi-Hit: Multiple mutations in the same gene within a sample. In-Frame Del/Ins: Deletion/insertion preserving the reading frame, may affect protein function. Splice Site: Mutation affecting mRNA splicing. Translation Start Site: Mutation at initiation codon, blocking translation and potentially generating neoantigens. (**C-D**) Kaplan-Meier survival curves for high- vs. low-risk groups based on somatic mutations. Differences were evaluated using the log-rank test.

To investigate the genomic features underpinning risk stratification, we compared somatic mutation profiles between high- and low-risk HCC patients. Oncoplot visualization revealed distinct patterns, with TP53 mutations occurring more frequently in the low-risk group (46%) than in the high-risk group (28%), including unique variants (Fig. 7A, B). TMB analysis further demonstrated that patients with low TMB had significantly longer survival (*p* = 0.020, Fig. 7C). However, no significant difference in TMB levels was observed between high- and low-risk groups (*p* = 0.31), indicating that TMB is not a primary driver of risk stratification by our gene signature (Supplementary Figure S4). When combined with the transcriptome-based risk score in a four-quadrant framework, prognostic resolution was sharpened: patients in the L-TMB + low-risk group showed the most favorable outcomes, whereas those in the H-TMB + high-risk group experienced the poorest prognosis (*p* < 0.001, Fig. 7D). These results underscore the synergistic prognostic impact of genomic alterations and transcriptional risk stratification, and suggest that L-TMB + low-risk patients may derive the greatest benefit from immunotherapy, while H-TMB + high-risk patients likely require intensified therapeutic strategies. To assess potential clinical utility, we evaluated drug sensitivity using the GDSC database with the pRRophetic algorithm. Comparative analysis identified agents with differential predicted efficacy across risk groups (Fig. 7E). High-risk patients were predicted to be more sensitive to Tozasertib, WEHI-539, PAK_5339, GDC0810, ZM447439, WIKI4, MK-1775, Navitoclax, and BPD-00008900, as reflected by lower IC50 values, whereas SB505124 was predicted to be more effective in low-risk patients. Together, these findings demonstrate that the six-gene risk score not only stratifies prognosis but also captures distinct genomic and pharmacologic vulnerabilities, providing a potential framework for precision therapeutic decision-making in HCC.

### Validation of core prognostic genes by immunohistochemistry

**Fig. 8:**
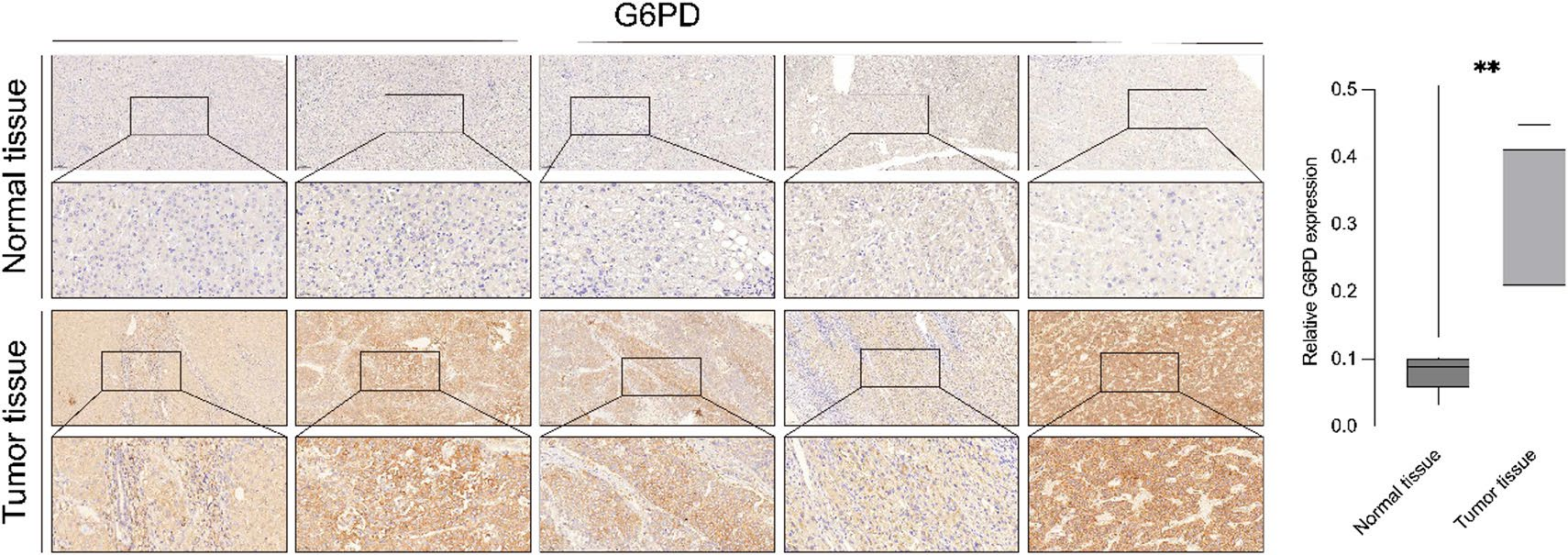
Immunohistochemical staining and quantitative analysis of G6PD expression in HCC tissues. (**A**) Representative IHC images showing G6PD protein expression in paired normal (upper panel) and tumor (lower panel) liver tissues at low (top) and high (bottom) magnification. Brown staining indicates positive G6PD expression. (**B**) Box plot showing significantly higher G6PD expression in tumor tissues compared to normal tissues (*p* < 0.01).

To further substantiate our transcriptomic findings at the protein level, IHC validation was performed for all six core prognostic genes identified in this study. Among them, G6PD, a rate-limiting enzyme of the pentose phosphate pathway critically involved in cellular redox homeostasis and metabolic adaptation, was selected as a representative gene due to its strong prognostic relevance and consistent expression pattern across cohorts. The IHC analysis was conducted on a cohort of ten non-cancerous liver tissues, including five adjacent non-tumor samples and five histologically normal liver specimens. Consistently, we observed markedly enhanced cytoplasmic staining of G6PD in tumor sections, whereas non-tumor tissues showed only weak or negligible staining. Quantitative scoring of staining intensity (H-score) confirmed that G6PD expression was significantly higher in tumor tissues compared with controls (*p* < 0.01). Representative images are shown in Fig. 8. In addition, IHC staining for the remaining five core prognostic genes was also performed, and consistent protein-level expression trends were observed between tumor and non-tumor tissues. These results further support the reliability of the prognostic signature. The corresponding IHC images and quantitative analyses for these five genes are provided in Supplementary Figure S5.

## Discussion

In this study, we established and validated a prognostic model for HCC that integrates transcriptomic, genomic, and pharmacogenomic features, thereby providing a multidimensional perspective on tumor biology and therapeutic vulnerabilities^22,23^. Our findings highlight that the candidate biomarkers identified not only discriminate patient prognosis with high accuracy but also embody mechanistic links to immune modulation, genomic instability, and drug response, underscoring their translational potential in precision oncology^24^. The robust prognostic performance of the gene signature across internal and external validation cohorts emphasizes its generalizability. Importantly, the functional annotation of prognostic biomarkers revealed consistent enrichment in pathways central to tumor progression, including cell cycle regulation, metabolic reprogramming, and DNA damage response^22,25,26^. These biological processes have long been implicated in HCC pathogenesis, yet our results provide a refined, biomarker-guided lens to interrogate their role in determining clinical outcomes^27^. Notably, the immune infiltration analysis uncovered distinct immune landscapes between risk groups, suggesting that transcriptional biomarkers may serve as surrogates of tumor-immune crosstalk^28,29^. In particular, low-risk patients exhibited enrichment of cytotoxic T cell signatures and higher immune activation scores, potentially reflecting an immune-permissive microenvironment conducive to favorable outcomes^30–32^. Conversely, high-risk tumors were characterized by immune exhaustion phenotypes, aligning with prior reports that link dysfunctional T cell infiltration to resistance against immune checkpoint inhibitors^33^. Furthermore, our IHC validation provided orthogonal evidence at the protein level. The differential expression patterns of representative biomarkers between high- and low-risk groups were consistent with transcriptomic predictions, thereby reinforcing the biological relevance of our model. Importantly, the IHC results further confirmed that high-risk tumors exhibited stronger expression of proliferation- and invasion-associated proteins, whereas low-risk tumors were enriched for markers indicative of immune activation.

Our integrative analysis of somatic mutations further supports the biological plausibility of the prognostic model. The elevated TMB observed in specific risk groups may contribute to altered neoantigen landscapes, thereby shaping immune recognition and therapeutic responsiveness^34,35^. Moreover, the identification of distinct mutational signatures and recurrently mutated genes highlights the genomic heterogeneity underpinning prognostic divergence in HCC^36^. These findings reinforce the concept that transcriptional and mutational features are not independent layers but are interconnected dimensions of tumor evolution and clinical behavior. Beyond biological insights, our pharmacogenomic predictions hold clinical implications. By leveraging the GDSC dataset and pRRophetic modeling, we uncovered candidate therapeutic agents with differential sensitivity between risk groups^37^. This strategy bridges the gap between prognostic stratification and actionable interventions, suggesting that our model may guide not only outcome prediction but also personalized therapy selection^38^. In particular, drugs with lower predicted IC50 values in the high-risk cohort could offer novel therapeutic opportunities for patients with dismal prognosis, thereby addressing a critical unmet clinical need^39^. While these predictions require rigorous experimental validation, they provide a rational starting point for drug repurposing and precision trial design. Several aspects of our findings warrant discussion in the context of the broader literature^40,41^. First, the immune relevance of our biomarkers resonates with growing evidence that HCC is an immune-sensitive tumor. Recent clinical successes of immune checkpoint inhibitors underscore the need for biomarkers that stratify patients according to immune responsiveness^42–44^. Our results suggest that transcriptional risk models, when coupled with immune infiltration analysis, may refine patient selection for immunotherapy beyond conventional markers such as PD-L1 expression or TMB. Second, the integration of somatic mutation profiling with prognostic modeling provides a mechanistic framework to reconcile inter-patient heterogeneity. This approach aligns with the emerging paradigm of multi-omic prognostic models, which promise to outperform single-modality predictors in complex cancers.

To further benchmark the prognostic performance of our gene signature, we conducted direct comparisons with several published HCC prognostic models using the same training cohort (model_1: PMID 32297074; model_2: PMID 34975338; model_3: PMID 39974411; model_4: PMID 39408693). The time-dependent ROC analysis revealed that our model achieved AUC values of 0.805, 0.709, and 0.702 for 1-, 3-, and 5-year overall survival predictions, respectively, which were comparable to or exceeded those of the comparator models. Particularly for 1-year survival prediction, our signature exhibited higher discriminative ability (AUC = 0.805) compared with model_2 (AUC = 0.709), model_3 (AUC = 0.730), and model_4 (AUC = 0.710), suggesting potential advantages in early risk stratification. Although model_1 showed relatively stable performance in 3- and 5-year predictions, the overall prognostic discrimination of our model remained similar or superior across key time points. These comparative analyses demonstrate that the gene signature not only robustly stratifies HCC prognosis but also performs favorably against existing prognostic signatures in the literature (Supplementary Figure S6).

Nevertheless, our study has limitations that should be acknowledged. The retrospective nature of the analyses, predominantly based on public datasets, introduces potential biases related to sample collection, processing, and clinical annotation^45^. Although external validation was performed, prospective clinical validation in large, ethnically diverse cohorts is necessary to confirm the clinical utility of the prognostic model^46^. Additionally, the pharmacogenomic predictions, while statistically robust, remain computational estimates and require functional validation in preclinical models and clinical settings. Finally, while our analysis uncovered associations between biomarkers and immune infiltration, causality cannot be inferred. Dissecting the mechanistic role of these genes in shaping tumor-immune interactions will require experimental perturbation studies^47^.

Looking forward, our findings lay the groundwork for several research directions. Integration of spatial transcriptomics and single-cell sequencing could delineate the spatial architecture of immune infiltration in HCC, providing a more granular understanding of the tumor ecosystem. Functional studies investigating the causal role of candidate biomarkers in immune modulation, metabolic reprogramming, or genomic instability will be crucial for mechanistic validation. Clinically, prospective trials embedding biomarker stratification into treatment decision-making could accelerate translation into precision oncology practice. Ultimately, the convergence of multi-omic profiling, computational modeling, and clinical validation holds the promise of transforming prognostic models from research tools into clinically actionable instruments that shape therapeutic strategies in HCC and beyond.

## Conclusion

This study demonstrates that integrative multi-omic approaches can yield prognostic models with both biological depth and clinical applicability. By linking transcriptomic biomarkers to immune landscapes, mutational processes, and drug sensitivities, we provide a holistic framework for understanding HCC heterogeneity and tailoring patient management. These insights contribute to the ongoing endeavor to advance precision oncology in liver cancer, with the overarching goal of improving patient outcomes through individualized prognostication and therapy.

## Supplementary Information

Below is the link to the electronic supplementary material.

Supplementary Material 1

Supplementary Material 2

Supplementary Material 3

Supplementary Material 4

Supplementary Material 5

Supplementary Material 6

Supplementary Material 7

## Data Availability

The RNA-seq and matched clinical data for hepatocellular carcinoma analyzed in this study are publicly available from TCGA via the GDC Data Portal (TCGA-LIHC: https://portal.gdc.cancer.gov/) and from the GEO database under accession number GSE112790 (https://www.ncbi.nlm.nih.gov/geo/query/acc.cgi?acc=GSE112790). The curated list of DRGs used in this work was extracted from previously published literature. Additional processed data, custom analysis scripts and code supporting the findings of this study are available from the corresponding author upon reasonable request.
