# Supplementary figures and images for: A demethylation-driven gene signature predicts prognosis and therapeutic vulnerability in hepatocellular carcinoma
Source: Sci Rep. 2026 Feb 26;16:11170. doi: 10.1038/s41598-026-41443-0 (PMC13046747; doi:10.1038/s41598-026-41443-0)

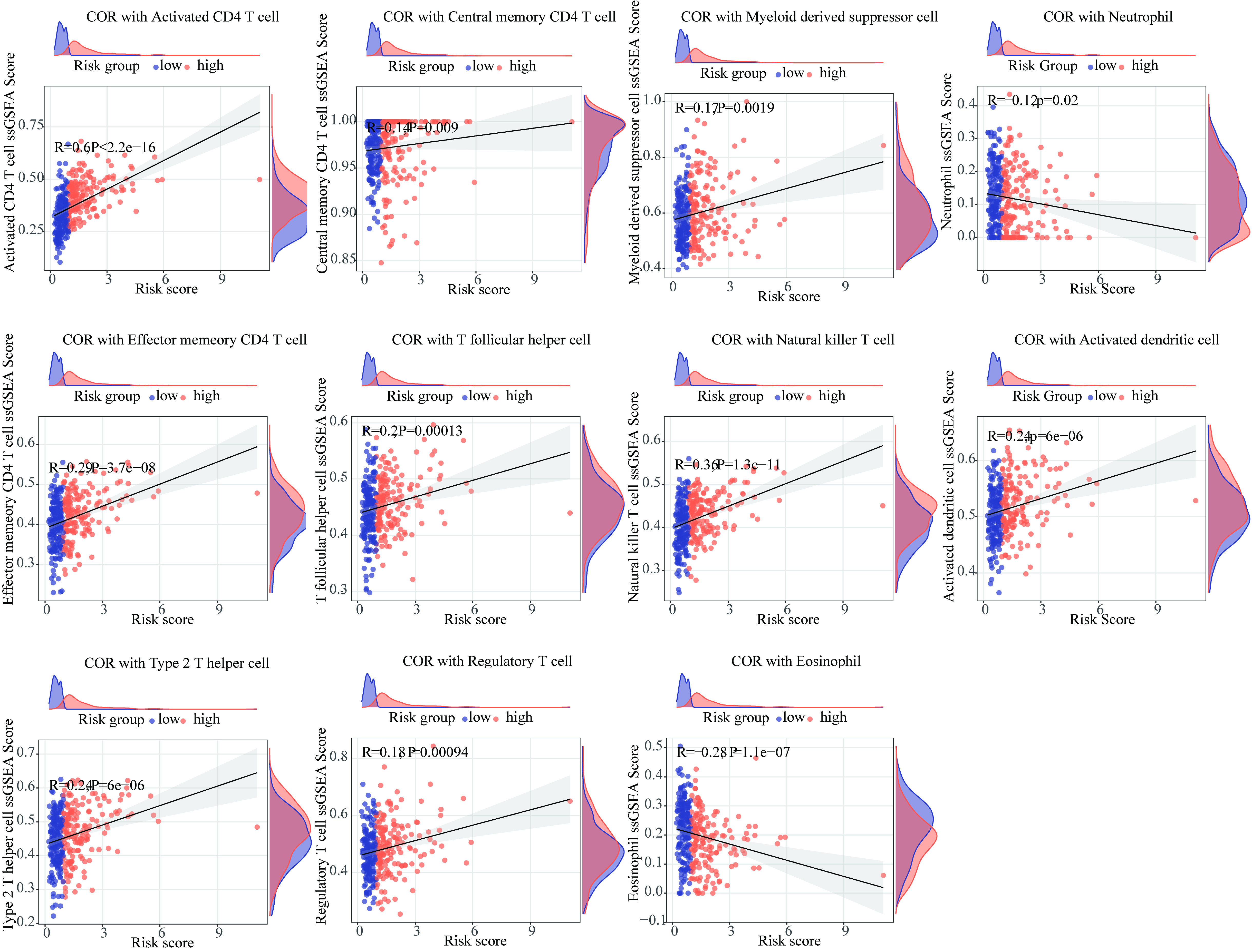

Supplement: Supplementary file 2 — Supplementary Material 2 [file 41598_2026_41443_MOESM2_ESM.tif]

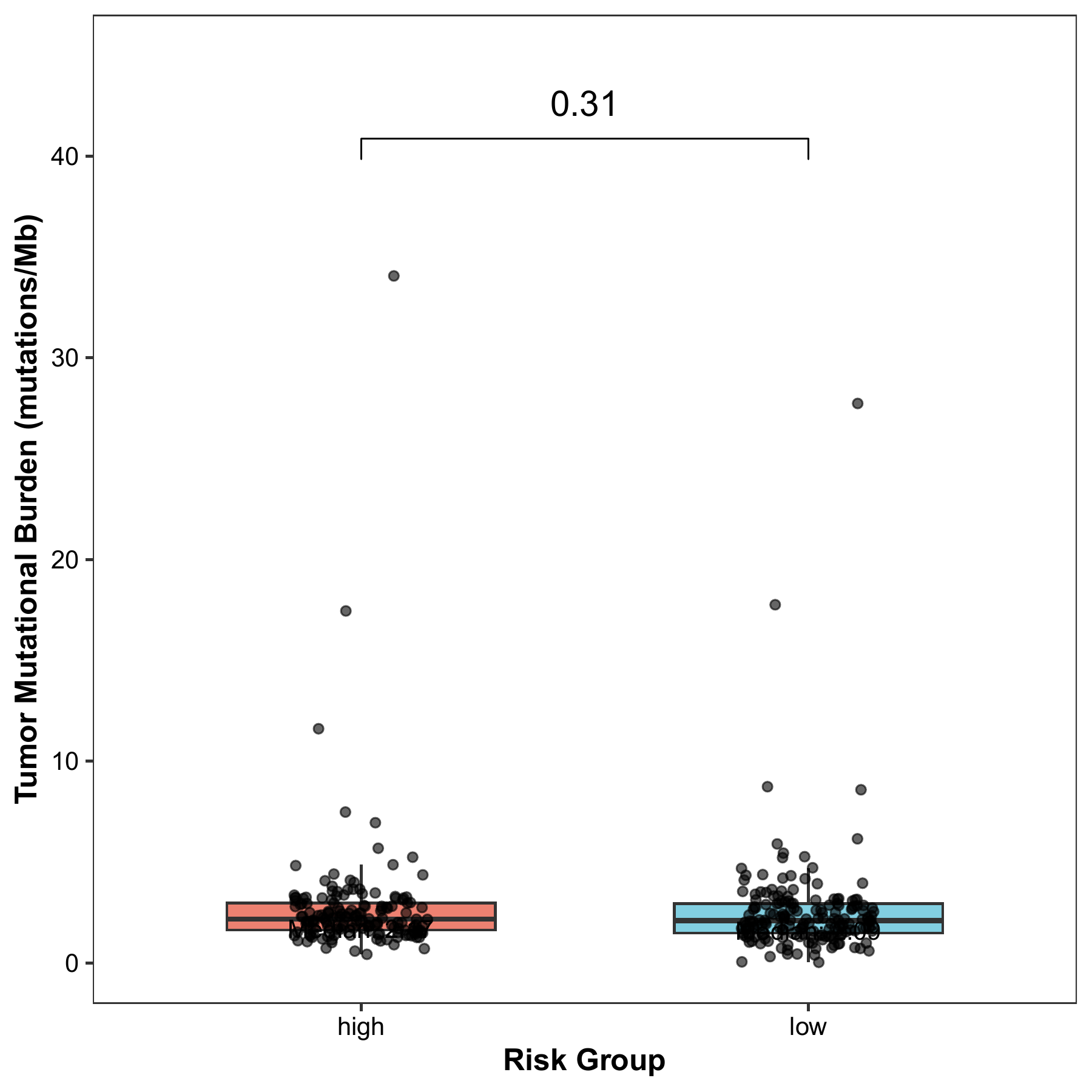

Supplement: Supplementary file 3 — Supplementary Material 3 [file 41598_2026_41443_MOESM3_ESM.tiff]

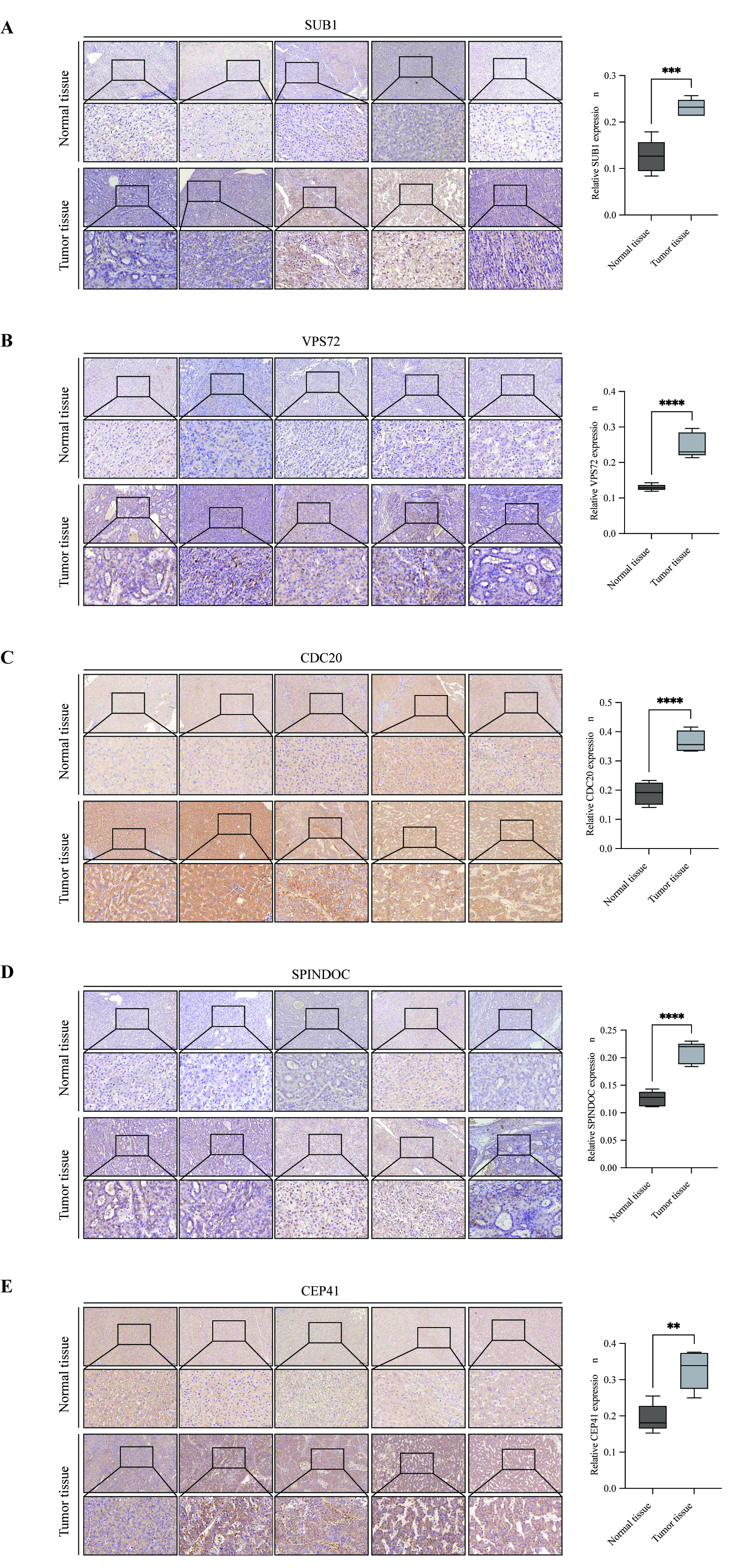

Supplement: Supplementary file 4 — Supplementary Material 4 [file 41598_2026_41443_MOESM4_ESM.tif]

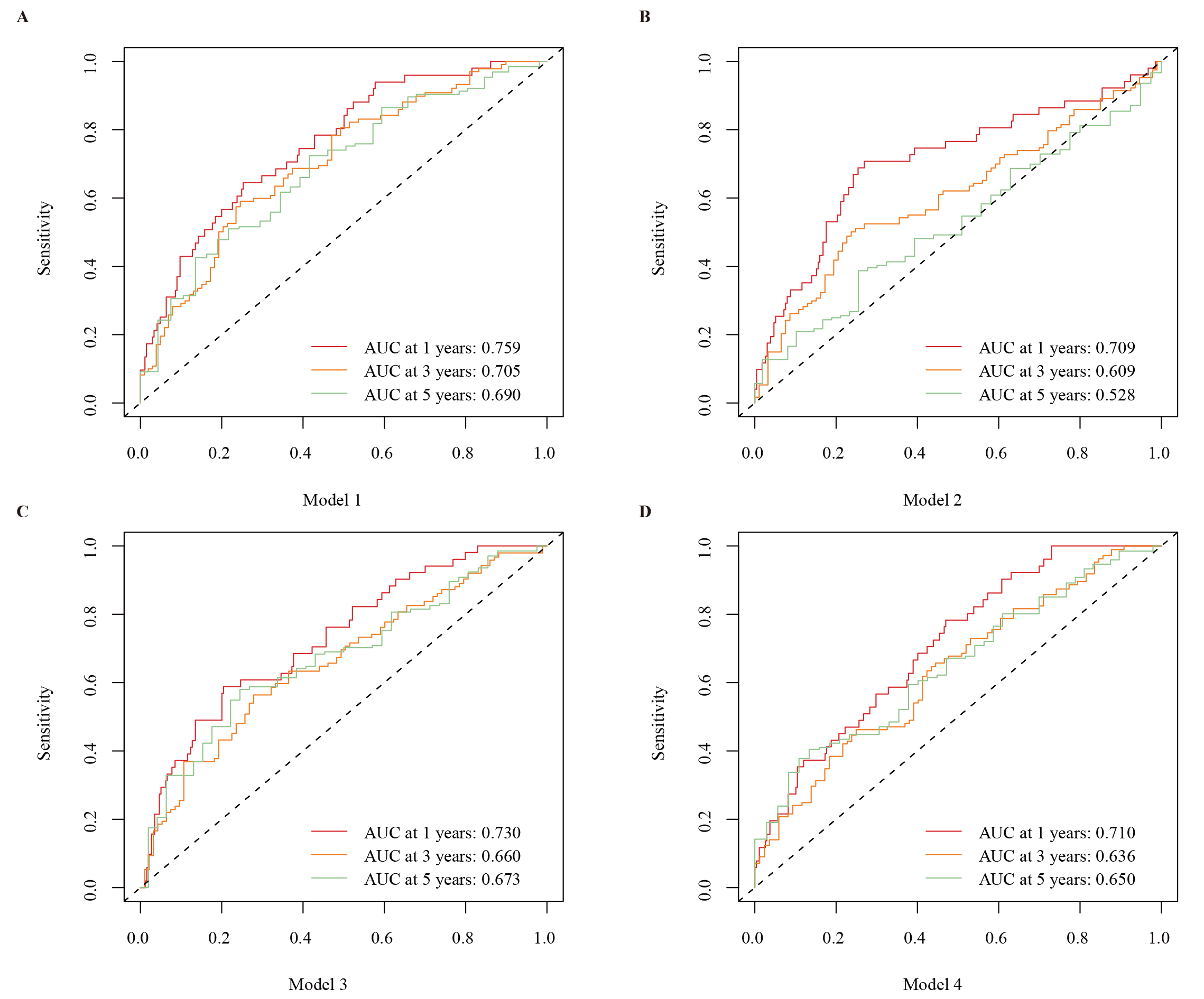

Supplement: Supplementary file 5 — Supplementary Material 5 [file 41598_2026_41443_MOESM5_ESM.tif]

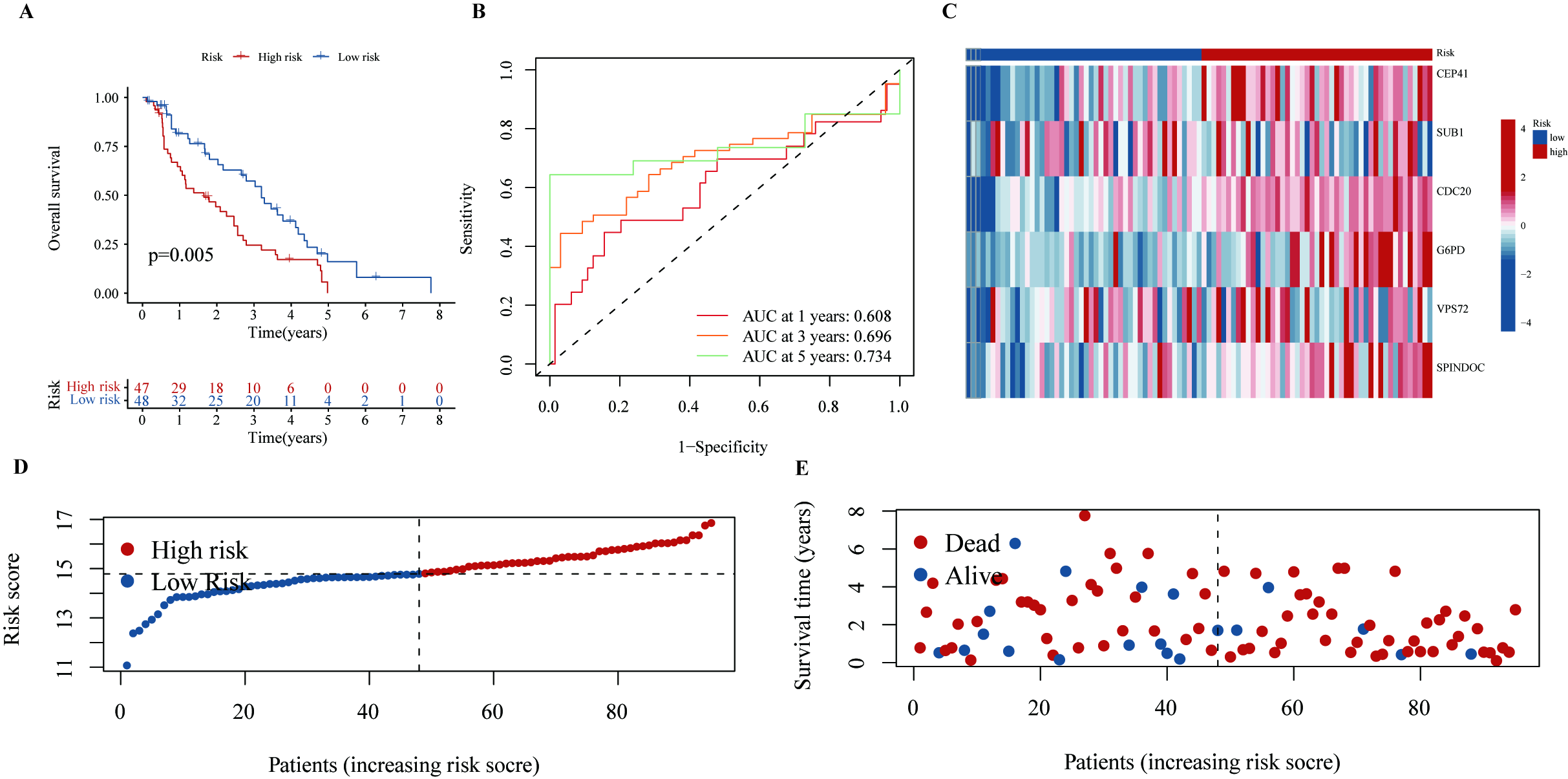

Supplement: Supplementary file 6 — Supplementary Material 6 [file 41598_2026_41443_MOESM6_ESM.tif]
